# Supplementary material for: Online psychoeducation and digital assessments as a first step of treatment for borderline personality disorder: A protocol for a pilot randomized controlled trial
Source: PLoS One. 2023 Dec 7;18(12):e0294331. doi: 10.1371/journal.pone.0294331 (PMC10703320; doi:10.1371/journal.pone.0294331)
Supplement: S2 File — (DOCX) [file pone.0294331.s002.docx]

| **Video topic** | **Clinician** | **Link** |
| --- | --- | --- |
| Self-Compassion | David Rosmarin, PhD, ABPP | <https://www.mcleanhospital.org/video/tap-power-self-compassion> |
| Depression | Christian Webb, PhD | <https://www.mcleanhospital.org/video/depression-101> |
| Coping | Lisa Coyne, PhD | <https://www.mcleanhospital.org/video/coping-uncontrollable> |
| Exercise | Marni Chanoff, MD | <https://www.mcleanhospital.org/video/supporting-healthy-mind-through-diet-exercise> |
| Diet | Marni Chanoff, MD | <https://www.mcleanhospital.org/video/supporting-healthy-mind-through-diet-exercise> |
| Relationships | Lisa Coyne, PhD | <https://www.mcleanhospital.org/video/maintaining-mentally-healthy-relationship> |
| Sleep | John Winkelman, MD, PhD | <https://www.mcleanhospital.org/video/good-nights-better-days-sleeps-relationship-mental-health> |
| Stress and Anxiety | Chris Palmer, MD | <https://www.mcleanhospital.org/video/your-everyday-guide-stress-management> |
| Self-care | Ana Trueba Yepez, PhD | <https://www.mcleanhospital.org/video/mental-benefits-self-care-regimen> |
| Self-acceptance | Lisa Coyne, PhD | <https://www.mcleanhospital.org/video/role-self-acceptance-good-mental-health> |

**Control videos**

**Note:** If desirable for replication, PI will provide the videos, controls (above), and platform if a research agreement can be made.
