# Supplementary material for: Online psychoeducation and digital assessments as a first step of treatment for borderline personality disorder: A protocol for a pilot randomized controlled trial
Source: PLoS One. 2023 Dec 7;18(12):e0294331. doi: 10.1371/journal.pone.0294331 (PMC10703320; doi:10.1371/journal.pone.0294331)
Supplement: S3 File — (DOCX) [file pone.0294331.s003.docx]

**Institutional Review Board**

**Intervention/Interaction Detailed Protocol**

Principal Investigator: Lois Choi-Kain, MD, MEd

Project Title: Psychoeducational videos and digital assessments for BPD

Version Date: 10/13/23

*For Intervention/Interaction studies, submit a Detailed Protocol that includes the following sections. If information in a particular section is not applicable, omit and include the other relevant information.*

1. **Background and Significance**

Borderline personality disorder (BPD) was once thought to be an untreatable personality organization, but it has since been legitimized as a diagnosis with multiple evidence-based treatments (EBT) (Gunderson, 2009). Many of these EBTs are intensive, long-term and have extensive training requirements for clinicians. These factors decrease scalability and contribute to the gap between supply and demand for BPD treatment (Iliakis et al., 2019; Choi-Kain et al., 2017).

Good Psychiatric Management (GPM) is a basic clinical management procedure that distills the fundamentals of how to treat any patient with BPD, starting with psychoeducation about the development, course, and treatment of the disorder and disclosure of the diagnosis (Gunderson & Links, 2014). GPM was shown to be non-inferior to Dialectical Behavior Therapy, a gold-standard specialized treatment for BPD, in a randomized control trial (McMain et al., 2009). Further research has supported psychoeducation as an effective intervention, independent of psychotherapeutic treatment. Zanarini et al. randomized 50 late adolescent women to attend a one day, in-person psychoeducational workshop about BPD in week 1 of the study or to a waitlist control condition. Participants in the workshop condition had significantly greater reductions in impulsivity and storminess of close relationships than participants in the waitlist condition (Zanarini et al., 2008). The same research group assessed the efficacy of a self-guided online psychoeducational program in a randomized control trial of 80 adult women with BPD and found that women in the treatment condition had a significantly greater reduction in all five sectors of borderline symptomatology studied (affective, cognitive, impulsivity, interpersonal, and over-all; Zanarini et al., 2017). Lastly, psychoeducation in a weekly group setting conducted over six sessions was tested against a waitlist control, and participants in the group had significantly greater reductions in affective, interpersonal, and cognitive symptoms, but not impulsivity (Ridolfi et al., 2019).

This study aims to test the feasibility and preliminary efficacy of psychoeducation delivered through a series of pre-recorded videos. Delivering the psychoeducation asynchronously avoids two significant sources of variability in treatment response: the therapist themself and the therapist’s role in the alliance (Wampold et al., 2005; Baldwin et al., 2007). This may be particularly helpful to individuals with BPD, who are hypersensitive in interpersonal relationships and interactions.

Our protocol also includes a rigorous battery of assessments, including neuropsychological testing with or without feedback, ecological momentary assessment (EMA), and traditional self-report measures. In addition to supporting a comprehensive view of participants’ change over time, this battery of assessments may also serve as an auxiliary intervention in itself. The EMA assessments may promote improved self-awareness, which has been shown to be impaired in adults with BPD (Pourmohammad et al., 2021). While there has been limited research regarding feedback on neuropsychological assessments, numerous published case studies have reported the benefits of feedback, which also increases self-awareness and allows support to be focused on areas of more severe impairment, while enhancing areas of higher ability (Finn, 2003; Gass & Brown, 1992; Gorske, 2008; Lopez et al., 2008; Malla et al., 1997). In addition, one non-randomized trial found that receiving feedback on neuropsychological assessment improved patient outcomes on metrics including quality of life and personal mastery (Rosado et al., 2018).

The aims of this study are significant for both patients and clinicians. Many individuals with BPD are unable to access care due to a lack of treatment options in their locale, stigma on the part of providers, or prohibitive costs in treatments not covered by insurance (Iliakis et al., 2019). Similarly, many primary care physicians (PCPs) are unable to provide referrals to appropriate treatment for their patients with BPD for the same reasons. A suite of online psychoeducational videos may augment treatment for a patient who is in a supportive therapy with a generalist clinician, or may be a first step in treatment that can be prescribed by a PCP.

1. **Specific Aims and Objectives**

**Specific Aims**

1. To test the feasibility and efficacy of a suite of 10 online educational videos about BPD as a scalable intervention to reduce BPD symptom severity.
2. To test the feasibility of an online assessment protocol that incorporates standard self-assessment and neuropsychological performance testing.
3. To test the feasibility and efficacy of brief, one-time neuropsychological feedback as an intervention to reduce BPD symptom severity.

**Hypotheses**

1. Participants who receive the suite of educational videos about BPD will have reduced symptom severity post-treatment compared to those who receive a suite of similar control videos without information about BPD.
   1. This effect will be mediated by increased knowledge about BPD.
   2. Differences in symptom severity between the BPD and non-BPD focused video conditions will be associated with measurable differences in self-reported symptoms and neuropsychological performance.
2. Participants who receive feedback will have reduced symptom severity post-treatment compared to those who do not receive feedback.
   1. This effect will be mediated by increases in consistency of self-report and social wisdom (i.e., learning) and decreases in social disconnection.
3. The educational videos and neuropsychological feedback will yield separate, measurable effects on emotional and interpersonal sensitivity in BPD.
4. **General Description of Study Design**

This study will follow a sequential multiple randomization design with two points of division: 1) psychoeducational or control videos, 2) feedback on neuropsychological assessments or no feedback. This yields three participant groups (the control/sham group will not undergo the second randomization):

- Psychoeducational videos x neuropsychological feedback
- Psychoeducational videos x no feedback
- Control/sham videos x no feedback

**Study Schema
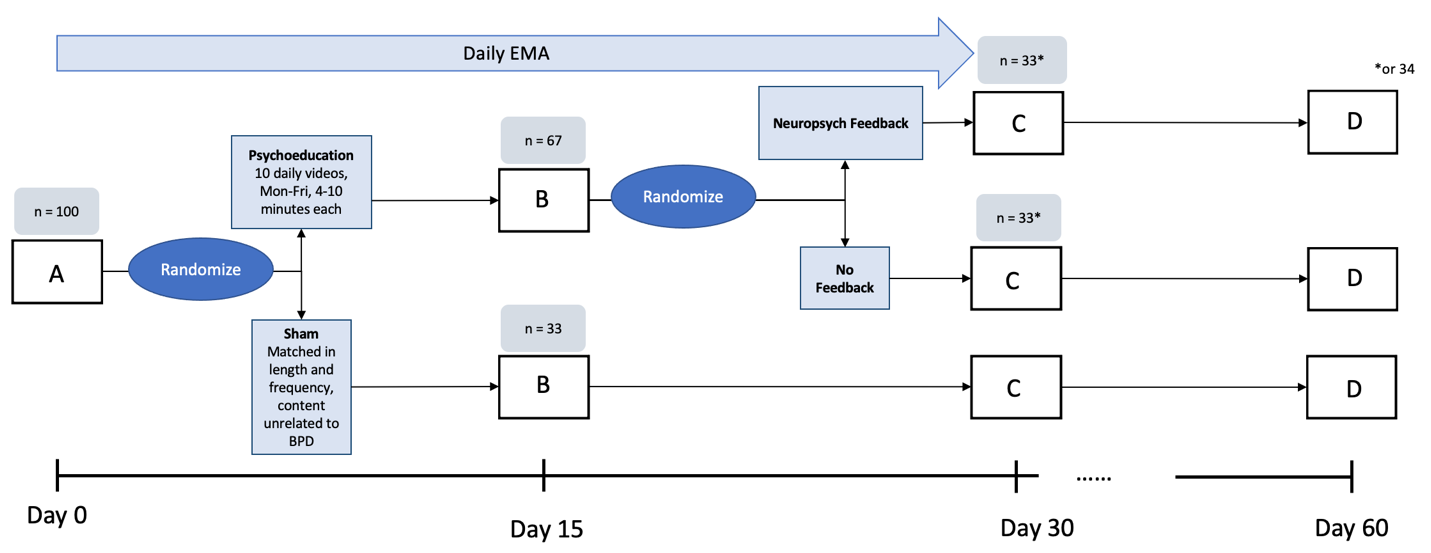
**

1. **Subject Selection**

**Inclusion Criteria:**

- Reliable access to a smartphone with a data plan for the duration of the study.
- Ability to speak and understand English.
- Age 18 years or older.
- Diagnosis of BPD within the past 6 months.
- Awake and able to complete EMA surveys between 9am-9pm on most days.
- Located within Massachusetts

**Exclusion Criteria:**

- Cognitive disability that impedes ability to participate in the study.
- Current psychiatric symptoms that interfere with the individual’s ability to provide consent or complete the research procedures (e.g., acute mania, acute psychosis, or eating disorders threatening medical stability).

**Recruitment Procedures:**

- The study coordinator (research assistant) is responsible for identifying and recruiting individuals.
- Individuals will be recruited on a rolling basis between April 2023 - May 2024, or until we reach our goal of 100 participants.
- Participants will be recruited in several ways. We will do some of the recruitment from units within McLean through designated research and clinical teams on the units. Potential participants meeting inclusion and exclusion criteria will be identified by embedded research assistants in the units and through Epic chart reviews by the study research assistant and PI. For participants identified through Epic, the study research assistant will contact the unit embedded RA and treatment team to ensure it is clinically appropriate to approach the patient for research. A unit staff member will ask the patient if they are interested in speaking with research staff. Capacity to consent or documentation of discussion of clinical appropriateness forms will be completed for all inpatients recruited.
- For the Behavioral Health Partial (BHP) program, treatment teams will obtain permission from patients to be contacted via email about a research study. BHP staff will then notify patients of the opportunity to participate in this research study via email (email template submitted to the IRB). Interested potential participants from the BHP will be the one to initiate contact with study staff. Study staff will then correspond with participants to set up a screening meeting.
- Study information will also be posted to the Mass General Brigham Rally platform and credible advocacy websites including the National Education Alliance for BPD in the form of a virtual flyer/post advertising the details of the study, a link to the virtual eligibility screening survey (see **Screening Questionnaire**), and the contact information of the study team (see **Advertisements**).
- Study information will be advertised with physical flyers posted in MGB-affiliated hospitals, as well as non-MGB hospitals including Boston Medical Center, UMass Memorial Medical Center, Beth Israel Deaconess Medical Center, Massachusetts Mental Health Center, UMass Amherst, Cambridge Health Alliance, and Tufts Medical Center.
- By advertising on the internet and in general healthcare settings to recently diagnosed individuals, we hope to mitigate the bias created by recruiting from subject pools who are already receiving specialized treatment, which is generally difficult to access (Iliakis et al., 2019).
- At branching points in the design, we will use block randomization to balance demographic and baseline variables between groups. Specifically, we will balance gender and BPD symptom severity at each randomization point.

1. **Subject Enrollment**

Participants will complete an online survey hosted on REDCap to determine their eligibility based on our inclusion and exclusion criteria. Eligible participants will be asked to meet with the PI or research assistant for a virtual consent discussion and will then sign a consent form.

Once enrolled in the study, all participants will fill out the baseline assessment battery, including demographic questions, the BPD Knowledge Test, Borderline Symptom List-23 (BSL-23), Patient Health Questionnaire-9 (PHQ-9), McLean Assessment of Rejection Sensitivity (MARS), Three-Item Loneliness Scale (LS-3), and the Level of Personality Functioning Scale - Brief (LPFS). They will then be randomized to receive the psychoeducational videos or the control videos using block randomization, considering demographic factors and baseline BPD symptoms (BSL-23). The randomization will be weighted such that 2/3 of participants (anticipated N=67) are in the BPD video condition and 1/3 (anticipated N=33) are in the non-BPD video condition. After completion of the first phase of the study, participants in the BPD psychoeducation arm will be randomly assigned to receive feedback or not. The second randomization will divide the participants equally, with an anticipated N=33 (or 34) in each arm.

1. **STUDY PROCEDURES**

**Outcomes:** The primary outcomes are borderline symptom severity (i.e., score on the Borderline Symptom Checklist) after each intervention point (timepoints B and C) and at the follow-up (timepoint D). The secondary outcomes are depressive symptoms severity (i.e., score on the Patient Health Questionnaire) after each intervention point (timepoints B and C) and at the follow-up (timepoint D).

**Screening**

Subjects will complete an online survey hosted on REDCap that includes a series of questions about their location, current psychiatric symptoms, availability, and smart phone access in order to assess their eligibility.

**Consenting**

After screening and before the first research assessment, eligible participants will complete the informed consent process. At the end of the screening survey, eligible participants will be asked to provide their contact information. The study staff will contact eligible participants to schedule a virtual consent discussion. The consent discussion will take place on an MGB Zoom account, and the Zoom link and consent form will be emailed to the participant in advance using secure MGB email. When participants present for the virtual consent discussion, the PI or study research assistant will fully explain the purpose of the research, the study procedures, the risks, discomforts, and possible benefits, and alternative existing treatments for BPD. The PI or research assistant will answer any questions from the participant. All participants in this study must be judged capable of understanding the nature of the study as well as the discomforts and potential benefits. The participants will be asked to return the signed consent form within 48 hours if they would like to participate, and the form will be signed by the PI or research assistant who completed the consent discussion. A fully signed copy of their consent form will be returned to the participant via secure email.

**Baseline Assessment (Timepoint A) - Day 1 (55 minutes)**

The baseline assessment battery includes a demographic questionnaire, neurocognitive tests, and self-report questionnaires regarding mental health and social functioning. The total length of the assessment is approximately 55 minutes.

- *Demographic Questionnaire:* Questions delivered through REDCap regarding participants’ sociodemographic information.
- *Self-Report Questionnaires:* These surveys are hosted on REDCap and will take about 25 minutes. The questionnaires are listed below.
- *Cognitive Tests:* Tests are hosted and administered on the digital research platform TestMyBrain.org. Participants will be automatically re-directed to TestMyBrain.org after completing the REDCap portion of the assessments. These tests take about 30 minutes in total. The measures are listed below, along with the self-report questionnaires.

Baseline Self-Reports and Cognitive Tests

**Self-Reports**

1. Treatment History Questionnaire (5 minutes)
   1. This questionnaire is adapted from the Background Information Schedule (*Zanarini MC: Background Information Schedule. Belmont, Mass, McLean Hospital, 1992*) It asks about the participant’s experiences with past forms of psychotherapy and pharmacological treatment.
2. BPD Knowledge Test (4 minutes)
   1. This questionnaire, developed for the study, assesses the participant’s knowledge about symptoms, basic facts, long term course, comorbidities, and treatment of BPD. There is no existing measure of BPD knowledge.
3. Borderline Symptom List (BSL-23) (4 minutes)
   1. Bohus M, Kleindienst N, Limberger MF, Stieglitz RD, Domsalla M, Chapman AL, Steil R, Philipsen A, Wolf M. The short version of the Borderline Symptom List (BSL-23): development and initial data on psychometric properties. Psychopathology. 2009;42(1):32-9.
   2. This questionnaire assesses the severity of BPD symptoms.
4. Patient Health Questionnaire (PHQ-9) (2 minutes)
   1. Kroenke K, Spitzer RL, Williams JB. The PHQ‐9: validity of a brief depression severity measure. Journal of general internal medicine. 2001 Sep;16(9):606-13.
   2. This questionnaire assesses depression symptoms.
5. McLean Assessment of Rejection Sensitivity (MARS) (2 minutes)
   1. This unpublished questionnaire assesses trait-level rejection sensitivity.
6. Three-Item Loneliness Scale (LS-3) (1 minute)
   1. Hughes ME, Waite LJ, Hawkley LC, Cacioppo JT. A short scale for measuring loneliness in large surveys: Results from two population-based studies. Research on aging. 2004 Nov;26(6):655-72.
   2. This questionnaire assesses the level of overall loneliness.
7. San Diego Wisdom Scale (SD-WISE) (5 minutes)
   1. Thomas ML, Bangen KJ, Palmer BW, Martin AS, Avanzino JA, Depp CA, Glorioso D, Daly RE, Jeste DV. A new scale for assessing wisdom based on common domains and a neurobiological model: The San Diego Wisdom Scale (SD-WISE). Journal of Psychiatric Research. 2019 Jan 1;108:40-7.
   2. This questionnaire assesses general wisdom.
8. Level of Personality Functioning Scale-Brief (LPFS) (4 minutes)
   1. Weekers LC, Hutsebaut J, Kamphuis JH. The Level of Personality Functioning Scale‐Brief Form 2.0: Update of a brief instrument for assessing level of personality functioning. Personality and Mental Health. 2019 Feb;13(1):3-14.
   2. This questionnaire assesses traits of personality disorders dimensionally, rather than diagnostically or categorically.

**Cognitive Tests**

1. Gradual Onset Continuous Performance Test (CPT) (6-minute average completion time)
   1. Participants see a series of city or mountain scenes. The participant is asked to press a button whenever they see a city scene and withhold a response whenever they see a mountain scene. This a test of sustained attention and response inhibition. https://testmybrain.org/tests/gradCPT/index_down_mobilized.php
2. Reading the Mind in the Eyes (RMET) (8-minute average completion time)
   1. Participants see a series of images showing only the eye region of someone’s face. The participant is asked to choose which of four complex emotion words describes the mental state of the person depicted in the image. This is a test of theory of mind and social understanding. https://www.testmybrain.org/tests/mind_in_eyes/
3. Belmont Emotion Sensitivity Test (BEST) (7.5-minute average completion time)
   1. Participants see a series of pairs of faces and are asked to indicate which one is expressing more of the target emotion (happiness, anger, or fear). This test measures face emotion perception. https://www.testmybrain.org/tests/emotion_comparison/anger.html
4. Digit Span Tests - Forward and Backward (DST) (7-minute average completion time)
   1. Participants are asked to recall sequences of digits of increasing length, either in the same order as presented (Forward condition) or in the opposite order (Backward condition). This test measures auditory span, short term memory, attention, and working memory (backward version). This test will be presented to participants in a similar format to the other cognitive tests, linked above. A demo link is not currently available for this test.
5. Digit Symbol Matching Test (DSMT) (2-minute average completion time)
   1. Participants are asked to use a symbol-number key shown on screen to match as many symbols and numbers as possible in 90 seconds. This test measures processing speed and visual short-term memory. This test will be presented to participants in a similar format to the other cognitive tests above.

**Ecological Momentary Assessment - Days 1-30 (6 minutes per day)**

EMA data will be collected over the course of 30 days. Participants will complete one assessment each day at a randomly generated time between the hours of 9:00am-9:00pm. The assessment will be delivered to the participant’s smart phone as a text alert. The text message will contain a link to a REDCap survey, which will then re-direct to TestMyBrain.org for completion of two brief cognitive tests (the brief version of the Gradual Onset Continuous Performance Test - 1-2 minutes and the Digit Symbol Matching Test – 1-2 minutes) and questions regarding the participant’s immediate surroundings, functioning, recent social interactions, as well as feelings of connectedness, threat, aloneness, and despair. For the full text of the EMA questions, see the **EMA** document.

EMA survey data will be collected through REDCap and stored in the secure REDCap database. The EMA cognitive test data will be collected through TestMyBrain.org and stored in the secure TestMyBrain database (see **Risks and Discomforts**). The cognitive tests and EMA questions will not be linked to any identifying participant information. Instead, the survey links will be unique to each participant and individualized with a coded ID. IP addresses will not be stored with the data, and after the cognitive test data has been downloaded from the TestMyBrain database, any study-specific information will be deleted from the database.

The text messages including the EMA survey links will be sent using REDCap and Twilio. Twilio is a cloud computing platform that enables text messaging from a website. The phone numbers, and any other patient health information, will not be stored in REDCap and will not be permanently stored in Twilio.

**Second Assessment (Timepoint B) - Day 15 (35 minutes)**

The second assessment battery includes repeat administrations of one neurocognitive test, and some self-report questionnaires regarding mental health and social functioning that were administered at baseline. The protocol for administering the tests and questionnaires is the same as at baseline.

1. DST
2. BPD Knowledge Test
3. BSL-23
4. PHQ-9
5. LS-3
6. LPFS
7. CSQ-8
   1. Larsen DL, Attkisson C, Hargreaves WA, Nguyen TD. Assessment of client/patient satisfaction: Development of a general scale. Evaluation and program planning. 1979;2:197-207.
   2. This questionnaire is a brief acceptability measure.
8. Hospitalization Check-In
   1. This brief, unpublished questionnaire assesses if the participant has visited the emergency department, been hospitalized, or started a residential treatment program since the previous time point.

**Third Assessment (Timepoint C) - Day 30 (30 minutes)**

The third assessment battery includes repeat administrations of some neurocognitive tests, and some self-report questionnaires regarding mental health and social functioning that were administered at baseline. The protocol for administering the tests and questionnaires is the same as at baseline.

1. CPT
2. DST
3. DSMT
4. BSL-23
5. PHQ-9
6. LS-3
7. LPFS
8. Hospitalization Check-In

**Follow-up Assessment (Timepoint D) - Day 60 (50 minutes)**

The fourth assessment battery includes repeat administrations of all neurocognitive tests and self-report questionnaires regarding mental health and social functioning that were administered at baseline. The protocol for administering the tests and questionnaires is the same as at baseline.

1. CPT
2. RMET
3. BEST
4. DST
5. DSMT
6. Treatment History Questionnaire
7. BPD Knowledge Test
8. BSL-23
9. PHQ-9
10. MARS
11. LS-3
12. SD-WISE
13. LPFS

**Schedule of Assessments**

| **Measure** | **Baseline (Time 1)**  **Day 1** | **Time 2**  **Day 15** | **Time 3**  **Day 30** | **Follow-up**  **Day 60** |
| --- | --- | --- | --- | --- |
| **Demographics** | X |  |  |  |
| **CPT** | X |  | X | X |
| **RMET** | X |  |  | X |
| **BEST** | X |  |  | X |
| **DST** | X | X | X | X |
| **DSMT** | X |  | X | X |
| **Treatment Hist.** | X |  |  | X |
| **BPD Knowledge** | X | X |  | X |
| **Hosp. Check-In** |  | X | X |  |
| **BSL-23** | X | X | X | X |
| **PHQ-9** | X | X | X | X |
| **MARS** | X |  |  | X |
| **LS-3** | X | X | X | X |
| **SD-WISE** | X |  |  | X |
| **LPFS** | X | X | X | X |
| **CSQ-8** |  | X |  |  |
| **EMA Measures** | *Daily from Day 1 - Day 30* | |  |  |

**Description of Intervention**

- A total of ten videos will be delivered daily Monday-Friday over approximately 2 weeks (days 1-15). The BPD-related videos will feature the PI, a mental health professional who is an expert in BPD, explaining the topic in relatable language that can be understood by the general public. Each video will be approximately 4-10 minutes long, for a total of ~82 minutes of video. Videos will be embedded in REDCap and shared via a hyperlink in a text message. Sham videos will be matched to the psychoeducational videos in length, frequency, and general format.
  - Psychoeducational Video Content: The psychoeducational videos will cover the following topics:
    - Symptoms of BPD
    - The interpersonal hypersensitivity model of BPD
    - Basic facts about BPD
    - Long term course of BPD
    - Co-occurring disorders
    - Key principles for recovery
    - Common factors across treatments
    - Pharmacotherapy for BPD
    - Psychotherapeutic treatment options for BPD
    - Review of top 10 tips to guide recovery
  - Sham Video Content: The sham videos will discuss aspects of mental and physical health that are not related to BPD. Topics include:
    - Depression 101
    - Self-care
    - Sleep
    - Relationships and mental illness
- In the second phase of the study, participants randomized to the Feedback condition will receive an email with a personalized summary of their symptom endorsement and neuropsychological performance. The feedback will include relevant assessment scores or sub-scores . For each score and sub-score, there will be a description of the cognitive domain that the metric assesses (e.g., attention span in the CPT). See the attached example for how feedback on each test will be formatted and phrased. We will tailor the feedback to each participant’s scores. We may adjust the format or wording of the feedback based on participant responses (i.e., if a participant alerts us that some wording is unclear or confusing). We may also alter the feedback if we learn new information about the relevance or interpretability of these cognitive tests, as this is a growing field of research.

**Remuneration**

Participants can receive a total of $100.00 if they complete all assessments in this study. The break-down of payments is as follows:

| $1 per daily assessment (30 total) | $ 30.00 |
| --- | --- |
| Timepoint A | $ 20.00 |
| Timepoint B | $ 15.00 |
| Timepoint C | $ 15.00 |
| Timepoint D | $ 20.00 |
| **Total** | **$ 100.00** |

At each timepoint, at least 50% of the questions included in the battery of questionnaires must be completed in order for the participant to qualify for payment for that specific timepoint. Payment will be delivered at the end of the study in a lump sum.

1. **Risks and Discomforts**

The amount of risk involved in this project is minimal. Participants are allowed to terminate their participation without penalty at any time. Participants also have the option to revoke their authorization for the researchers to collect and use their information, even after they have participated.

- **Privacy:** A potential risk is a breach of confidentiality. To protect against the loss of confidentiality, we will keep all physical study data locked in a separate file, coded by a unique study code number (with names and other personal identifying information kept separately). Electronic data will be encrypted, password protected and labeled with a participant ID number. Every participant is assigned a participant ID, and all participant data are connected only to that ID, not to a name, phone number, or email address.
- **Psychosocial Risks:** Self-Report Measures. Possible discomfort may occur while answering personal questions during the eligibility screening and self-report questionnaires. Efforts will be made to make the participants feel comfortable and at ease. Participants are allowed to terminate their participation without penalty at any time. Participants also have the option to revoke their authorization for the researchers to collect and use their information at any time. Cognitive Tests. Participants in the Feedback condition may not be comfortable with the results of their cognitive testing.
- **Data Charges:** Participants will use their personal smartphones to complete the assessments will be responsible for costs associated with their smartphone use. After each assessment that is completed, a small, encrypted data package is sent back to the study team. The amount of data is similar to one email containing only text, and even with daily transmissions, there should be no discernible impact on the participant’s data use. However, because they are sending data from their personal phones, if these transmissions cause them to exceed the size of their existing data plan, they may incur charges.

Every effort will be made to protect against potential risks associated with this study.

- **Protection against privacy risk:** Participant names or other personally identifying information will never be associated with any of the data. Computer workstations in the Gunderson Personality Disorder Institute offices will be used for data analysis and will be locked and password protected. All self-report and neuropsychological data will be coded with unique numbers and be temporarily stored in the Amazon Relational Database Service, a cloud-based data storage service connected to Amazon Web Services (i.e., TestMyBrain’s secure database) before being downloaded. After download, the data will be stored on study staff computers in locked offices. Access to all data will be limited to IRB-approved study staff.
- **Protection against psychosocial risk:**
- Participants will be told they can terminate their participation at any time if they become uncomfortable.
- All self-report assessments will include a reminder for patients that study staff will not see the data immediately and that if they feel that they need immediate attention, they should call 911 or go to their local Emergency Department. If a patient should contact the study staff directly and indicate that they feel at risk of hurting themselves, the following steps will be taken. First, the study staff member who received the message will advise the patient to call 911, go to their local Emergency Department, or call their outpatient treater, if applicable. The study staff member who received the message will then contact the principal investigator (PI), Dr. Choi-Kain. The PI will contact the participant as soon as possible and conduct a suicide risk assessment to determine whether it is necessary to take immediate action to prevent the participant from causing harm to him/ herself. If needed, actions include sending an ambulance so that the individual may be evaluated for inpatient psychiatric admission. If the study participant has experienced significant deterioration but is not in immediate danger of hurting him or herself, we will take the following actions. First, we will inform the patient about procedures for contacting emergency services should they find themselves at risk for self-harm. Second, if the patient wishes to access psychotherapy or psychiatric referrals, we will provide them.
- **Protection against data charges:** The study team will limit the data requirements as much as is feasible within the scope of the study. The planned data use is not projected to have an impact on the participant’s overall data consumption and is very unlikely to cause the participant to incur any charges.

1. **Benefits**

Participants in the Psychoeducational Video condition may benefit by learning more about the etiology, course, and treatment options for BPD. Participants in the Neuropsychological feedback condition may benefit by understanding their own symptom profile in more detail.

All participants may receive a benefit from direct experience with ongoing psychological research. Directly experiencing a psychology research project will give the participants an understanding of what type of procedures and practices are involved in the study of the human mind. They will also gain understanding of the scientific process; specifically, how these particular hypotheses are being tested. For some participants, the opportunity to participate in research and contribute to the understanding of psychiatric disorders can also provide a greater sense of meaning and sense of giving back to the community.

1. **Statistical Analysis**

We expect a small to medium effects on BPD symptom severity, in line with effect sizes in the meta-analytic review of BPD’s effective interventions. With a significance level of .05 and power of .8, the necessary sample size per condition to detect a small effect (Cohen’s *d =* .2) is 394, and to detect a medium effect (Cohen’s *d* = .4), *n* = 99. Although this pilot study is not adequately powered to detect the expected small to medium effect-sizes, we will conduct exploratory analyses in preparation for a future, large-scale investigation. We will 1) test associations between BPD symptoms and treatments in step 1 (video intervention) and step 2 (neuropsychological testing feedback) separately; 2) explore replacing binary treatment assignment with the actual number of educational videos one watched in a secondary analysis and test its association with BPD outcomes in step 1; 3) Mediation analysis to estimate the direct effect as well as the indirect effect, mediated by level of knowledge of BPD, of treatment to the BPD outcomes perform a moderator analysis; 3) perform longitudinal data analysis to examine the effects of different treatment combinations and 4) test whether differential responses to step 1 would be associated with changes in effectiveness of treatments in step 2.

1. **Monitoring and Quality Assurance**

- The PIs will have responsibility for continuous monitoring of data and safety of participants in the study. Data and safety monitoring will take place continuously throughout the study’s duration.
- In the unlikely event of adverse events or serious adverse events, these events will be documented and reported as required by the Partners Human Research Committees (PHRC) polity. As outlined by the PHRC policy, the principal investigator will report to the PHRC any of the following unanticipated problems and adverse events that occur: 1) during the conduct of the study, 2) after study completion, or 3) after participant withdrawal or completion. In such an event, reports will be submitted within 5 working days/7 calendar days of the date the investigator first becomes aware of the problem.
- At the time of the continuing review we will provide the Partners IRB with a summary of any unexpected and related adverse events as well as any other unanticipated problems that occurred since the last continuing review.

All other unanticipated incidents, experiences, information, outcomes, or other problems that indicate that the research places subjects at an increased risk of physical, psychological, economic, legal, or social harm than was previously known or recognized are to be submitted through Insight/eIRB as an Other Event.

The PI will ensure that the study adheres to the IRB-approved protocol and will frequently monitor the overall progress of the study. The study staff will ensure the accuracy and completeness of study materials and report all inconsistencies to the PIs.

1. **Privacy and Confidentiality**

Study procedures will be conducted in a private setting

Only data and/or specimens necessary for the conduct of the study will be collected

Data collected (paper and/or electronic) will be maintained in a secure location with appropriate protections such as password protection, encryption, physical security measures (locked files/areas)

Specimens collected will be maintained in a secure location with appropriate protections (e.g. locked storage spaces, laboratory areas)

Data and specimens will only be shared with individuals who are members of the IRB-approved research team or approved for sharing as described in this IRB protocol

Data and/or specimens requiring transportation from one location or electronic space to another will be transported only in a secure manner (e.g. encrypted files, password protection, using chain-of-custody procedures, etc.)

All electronic communication with participants will comply with Mass General Brigham secure communication policies

Identifiers will be coded or removed as soon as feasible and access to files linking identifiers with coded data or specimens will be limited to the minimal necessary members of the research team required to conduct the research

All staff are trained on and will follow the Mass General Brigham policies and procedures for maintaining appropriate confidentiality of research data and specimens

The PI will ensure that all staff implement and follow any Research Information Service Office (RISO) requirements for this research

Additional privacy and/or confidentiality protections

**12. References**

Baldwin SA, Wampold BE, Imel ZE. Untangling the alliance-outcome correlation: Exploring the relative importance of therapist and patient variability in the alliance. Journal of consulting and clinical psychology. 2007 Dec;75(6):842.

Bohus M, Kleindienst N, Limberger MF, Stieglitz RD, Domsalla M, Chapman AL, Steil R, Philipsen A, Wolf M. The short version of the Borderline Symptom List (BSL-23): development and initial data on psychometric properties. Psychopathology. 2009;42(1):32-9.

Choi-Kain LW, Finch EF, Masland SR, Jenkins JA, Unruh BT. What works in the treatment of borderline

personality disorder. Curr Behav Neurosci Rep. 2017;4(1):21-30.

Finn SE. Therapeutic Assessment of a man with" ADD". Journal of Personality Assessment. 2003 Apr 1;80(2):115-29.

Gass CS, Brown MC. Neuropsychological test feedback to patients with brain dysfunction. Psychological Assessment. 1992 Sep;4(3):272.

Gorske TT. Therapeutic neuropsychological assessment: A humanistic model and case example. Journal of Humanistic Psychology. 2008 Jul;48(3):320-39.

Gunderson JG. Borderline personality disorder: ontogeny of a diagnosis. American Journal of Psychiatry. 2009 May;166(5):530-9.

Gunderson JG, Links PS. Handbook of good psychiatric management for borderline personality disorder. American Psychiatric Pub; 2014 Jan 15.

Hughes ME, Waite LJ, Hawkley LC, Cacioppo JT. A short scale for measuring loneliness in large surveys: Results from two population-based studies. Research on aging. 2004 Nov;26(6):655-72.

Iliakis EA, Sonley AK, Ilagan GS, Choi-Kain LW. Treatment of borderline personality disorder: is supply

adequate to meet public health needs?. Psychiatr Serv. 2019;70(9):772-81.

Kroenke K, Spitzer RL, Williams JB. The PHQ‐9: validity of a brief depression severity measure. Journal of general internal medicine. 2001 Sep;16(9):606-13.

Larsen DL, Attkisson C, Hargreaves WA, Nguyen TD. Assessment of client/patient satisfaction: Development of a general scale. Evaluation and program planning. 1979;2:197-207.

Lopez C, Roberts ME, Tchanturia K, Treasure J. Using neuropsychological feedback therapeutically in treatment for anorexia nervosa: Two illustrative case reports. European Eating Disorders Review: The Professional Journal of the Eating Disorders Association. 2008 Nov;16(6):411-20.

Malla AK, Lazosky A, McLean T, Rickwood A, Cheng S, Norman RM. Neuropsychological assessment as an aid to psychosocial rehabilitation in severe mental disorders. Psychiatric Rehabilitation Journal. 1997;21(2):169.

McMain SF, Links PS, Gnam WH, Guimond T, Cardish RJ, Korman L, Streiner DL. A randomized trial of dialectical behavior therapy versus general psychiatric management for borderline personality disorder. american Journal of Psychiatry. 2009 Dec;166(12):1365-74.

Pourmohammad P, Imani M, Goodarzi MA, Sarafraz MR. Impaired complex theory of mind and low emotional self-awareness in outpatients with borderline personality disorder compared to healthy controls: A cross-sectional study. Journal of Psychiatric Research. 2021 Nov 1;143:445-50.

Ridolfi ME, Rossi R, Occhialini G, Gunderson JG. A clinical trial of a psychoeducation group intervention for patients with borderline personality disorder. The Journal of clinical psychiatry. 2019 Dec 31;80(1):0-.

Rosado DL, Buehler S, Botbol-Berman E, Feigon M, León A, Luu H, Carrión C, Gonzalez M, Rao J, Greif T, Seidenberg M. Neuropsychological feedback services improve quality of life and social adjustment. The Clinical Neuropsychologist. 2018 Apr 3;32(3):422-35.

Wampold BE, Brown GS. Estimating variability in outcomes attributable to therapists: a naturalistic study of outcomes in managed care. Journal of consulting and clinical psychology. 2005 Oct;73(5):914.

Weekers LC, Hutsebaut J, Kamphuis JH. The Level of Personality Functioning Scale‐Brief Form 2.0: Update of a brief instrument for assessing level of personality functioning. Personality and Mental Health. 2019 Feb;13(1):3-14.

Zanarini MC: Background Information Schedule. Belmont, Mass, McLean Hospital, 1992.

Zanarini MC, Frankenburg FR. A preliminary, randomized trial of psychoeducation for women with borderline personality disorder. Journal of personality disorders. 2008 Jun;22(3):284-90.

Zanarini MC, Conkey LC, Temes CM, Fitzmaurice GM. Randomized controlled trial of web-based psychoeducation for women with borderline personality disorder. The Journal of clinical psychiatry. 2017 Jul 11;78(3):0-.
